# Evaluation of Outcomes and Quality of Care in Children with Sickle Cell Disease Diagnosed by Newborn Screening: A Real-World Nation-Wide Study in France

**DOI:** 10.3390/jcm8101594

**Published:** 2019-10-02

**Authors:** Valentine Brousse, Cécile Arnaud, Emmanuelle Lesprit, Béatrice Quinet, Marie-Hélène Odièvre, Maryse Etienne-Julan, Cécile Guillaumat, Gisèle Elana, Marie Belloy, Nathalie Garnier, Abdourahim Chamouine, Cécile Dumesnil, Mariane De Montalembert, Corinne Pondarre, Françoise Bernaudin, Nathalie Couque, Emmanuelle Boutin, Josiane Bardakjian, Fatiha Djennaoui, Ghislaine Ithier, Malika Benkerrou, Isabelle Thuret

**Affiliations:** 1 Department of General Pediatrics and Pediatric Infectious Diseases, Sickle Cell Disease Reference Center, Necker-Enfants Malades Hospital, Assistance Publique-Hôpitaux de Paris (AP-HP), Université de Paris, 75005 Paris, France; 2 Department of Pediatrics, Sickle Cell Disease Reference Center, CHIC Hospital, Université de Paris-Est Créteil, 94000 Créteil, France; 3 Department of Pediatrics, Sickle Cell Disease Reference Center, Trousseau Hospital, Assistance Publique-Hôpitaux de Paris (AP-HP), 75012 Paris, France; 4 Department of Pediatrics, Louis Mourier Hospital, Assistance Publique-Hôpitaux de Paris (AP-HP), 92700 Colombes, France; 5 Sickle Cell Disease Unit, Sickle Cell Disease Reference Center, University Hospital of Pointe-à-Pitre/Abymes, BP 465 Pointe-à-Pitre, Guadeloupe, France; 6 Department of Pediatrics, Centre Hospitalier Sud Francilien, 91100 Corbeil-Essonne, France; 7 Sickle Cell Disease Unit, Sickle Cell Disease Reference Center, University Hospital of Martinique, 97261 Fort De France, Martinique, France; 8 Department of Pediatrics, Robert Ballanger Hospital, 93600 Aulnay Sous Bois, France; 9 Department of Pediatric Onco-Hematology, Institut d’Hématologie et d’Oncologie Pédiatrique, 69008 Lyon, France; 10 Department of Pediatrics, Mamoudzou Hospital, 97600 Mayotte, France; 11 Department of Pediatric Onco-Hematology, Charles Nicolle Hospital, 76600 Rouen, France; 12 Biochemistry and Molecular Biology Laboratory, Robert Debré Hospital, Assistance Publique-Hôpitaux de Paris (AP-HP), 75019 Paris, France; 13 Department of Public Health And Biostatistics, Henri Mondor Hospital, Assistance Publique-Hôpitaux de Paris (AP-HP), 94010 Créteil, France; 14 Department of Biochemistry and Genetics, Henri Mondor Hospital, Assistance Publique-Hôpitaux de Paris (AP-HP), 94010 Créteil, France; 15 Clinical Research Unit, Albert Chenevier Hospital, 94010 Créteil, France; 16 Sickle Cell Disease Reference Center, Robert Debré Hospital, Assistance Publique-Hôpitaux de Paris (AP-HP), 75019 Paris, France; 17 Department of Pediatric Onco-Hematology, Thalassemia Reference Center, Timone Enfant Hospital, Assistance Publique-Hôpitaux de Marseille (AP-HM), 13005 Marseille, France; Isabelle.thuret@ap-hm.fr

**Keywords:** sickle cell disease, newborn screening, mortality, morbidity, transcranial Doppler, vaccination coverage

## Abstract

This study’s objective was to assess, on a national scale, residual risks of death, major disease-related events, and quality of care during the first five years in children diagnosed at birth with sickle cell disease (SCD). Data were retrospectively collected from medical files of all children with SCD born between 2006–2010 in France. Out of 1792 eligible subjects, 1620 patients (71.8% SS or S/beta°-thalassemia -SB°-) had available follow-up data, across 69 centers. Overall probability of survival by five years was 98.9%, with 12/18 deaths related to SCD. Probability of overt stroke by five years in SS/SB° patients was 1.1%, while transcranial Doppler (TCD) was performed in 81% before three years of age. A total of 26 patients had meningitis/septicemia (pneumococcal in eight cases). Prophylactic penicillin was started at a median age of 2.2 months and 87% of children had received appropriate conjugate pneumococcal vaccination at one year. By five years, the probability of survival without SCD-related events was 10.7% for SS/SB° patients. In contrast, hydroxyurea was prescribed in 13.7% and bone marrow transplant performed in nine patients only. In this study, residual risks of severe complications were low, probably resulting from a good national TCD, vaccination, and healthcare system coverage. Nonetheless, burden of disease remained high, stressing the need for disease-modifying or curative therapy.

## 1. Introduction

Newborn screening (NBS) programs for sickle cell disease (SCD) aim to reduce early mortality and morbidity by introducing preventive measures directed towards major disease-related identified risks: Pneumococcal infection, acute splenic sequestration, and neurovascular injury. Prophylactic intervention such as penicillin and pneumococcal vaccination, parental education, and neurological screening by transcranial Doppler (TCD) have demonstrated significant efficacy in reducing these risks in children [1,2,3,4]. Recent studies from either monocentric reference centers [5,6] or regional multicenter hospital-based network [7] or performed on a national scale [8] in different high-income settings (USA, England, and France) have consistently shown low childhood mortality and an improved survival into adulthood of 94%–100% for SCD children diagnosed at birth and who benefitted from such measures.

Only very few nation-wide data are available, however, regarding both medical care and residual early morbidity and mortality in countries where newborn screening and prophylactic measures are implemented. Only such studies may in fact address disparities across regions, centers, or countries and provide a true-to-life evaluation.

A SCD NBS program was fully implemented since 2000 in France, targeting newborns identified at risk in mainland France and all newborns in French overseas territories. More specifically, regarding the targeted SCD NBS program, newborns are screened when parents originate from a country where SCD is endemic (an official list is available) and/or in case of family history of SCD and/or in case of uncertainty regarding both previous issues, in accordance with national guidelines [9]. Following confirmed diagnosis, children with SCD have free medical care regarding all SCD-related specific prophylactic or therapeutic measures, consultation, and hospitalization. A specialized follow-up is offered in the closest hospital with expertise in SCD, in relation to a tertiary reference center for additional expertise if needed. In addition, free medical surveillance and immunization is offered in France to all children aged less than six years.

The main objective of this retrospective study was to assess, on a national scale and during the first five years of life, residual risks of death, overt stroke, and bacterial meningitis/septicemia resulting from current transcranial Doppler (TCD) and pneumococcal prophylactic utilization in SCD children diagnosed at birth. The secondary objective was to evaluate in these young children the frequency of other main SCD-related events and the use of disease-modifying or curative therapy (hydroxyurea (HU), transfusion (TF) programs, and hematopoietic stem cell transplantation (HSCT)).

## 2. Experimental Section

### 2.1. Study Population

We retrospectively identified all patients born between 1 January 2006 and 31 December 2010 diagnosed with SCD through the national NBS program in mainland France, French West Indies (Guadeloupe, Martinique), and Réunion-Mayotte. Results of NBS indicating SCD were fed back to a referral sickle cell center where confirmatory tests were performed, and results were explained to parents. The follow-up was thereafter organized in the closest sickle cell center according to the child’s residence. Sickle cell centers were formally identified in a national network in 2004. Patients diagnosed in French Guyana were not included in the study because at the time of data collection, cross identification of patients following NBS was not possible in that region.

### 2.2. Data Collection

Data were collected in 2014/2015 from the patients’ medical files up to the age of 5 years regarding age at first prescription of penicillin prophylactic therapy, occurrence of death/overt, stroke/bacterial meningitis or septicemia, first event of acute splenic sequestration, acute chest syndrome/pneumonia, vaso-occlusive crisis (VOC), age at first transfusion, as well as use of pneumococcal vaccines/first chronic TF program/hydroxyurea/hematopoietic stem cell transplantation and TCD results.

Patients were considered lost to follow-up when no data were available during the 2-year period of data collection i.e., 2014/2015 in any center in France. In addition, patients residing abroad and who were seen only occasionally in France were excluded from analysis.

### 2.3. Outcomes

Fatal cases were analyzed in detail and the numbers were checked with the National French Death Registry for exhaustivity purpose.

TCD velocities were considered abnormal when ≥2 m/s and conditional when ≥1.7 m/s but <2 m/s (according to the STOP criteria [10]), as recorded in the medical files.

Other clinical outcomes were collected as reported in the medical file and no further verification was performed. However, the following definitions are consensual in France:

- Vaso-occlusive crisis (including dactylitis) was defined as an acute non-infectious, non-traumatic pain requiring analgesics for more than 12 h and/or hospital admission.
- Acute chest syndrome (ACS) was defined as a new pulmonary infiltrate on chest X-ray, with or without pain, cough, fever (≥38.5 °C), or hypoxemia. Given the overlapping definition of pneumoniae in young children, the latter events were pooled with ACS.
- Acute anemic events were defined by reductions in hemoglobin ≥20% versus steady state.
- Acute splenic sequestration (ASS) was defined as splenic enlargement increased ≥2 cm from baseline associated with acute anemia.
- Stroke was defined as an ischemic or hemorrhagic event lasting >24 h and resulting in focal neurologic deficit.

During the study period, national guidelines recommended prophylactic penicillin to all children with SCD at least until the age of 5, pneumococcal vaccinations using conjugate vaccines (3 injections of 7 valent pneumococcal conjugated vaccine (PCV7) in the first year of life (replaced by 13 valent pneumococcal conjugated vaccine (PCV13) from April 2010) followed by a boost during 2nd year, and a dose of polysaccharidic 23 valent pneumococcal vaccine (P23) at 2 years. Annual TCD screening was recommended from 2 to 16 years in patients with SS or SB°-thalassemia (SB°) disease only [11] and was performed with the same methodology across centers.

Chronic transfusion program was defined as any transfusion program that lasted >3 months. Hydroxyurea was recommended by national guidelines in 2005 for adults and children over 2 years with recurrent vaso-occlusive crises and/or acute chest syndrome but was specifically licensed with adapted pediatric dosage for patients with SCD in 2007 only.

### 2.4. Ethics

This study (NCT 03119922) was approved by the Inserm Ethics Committee/Institutional Review board (13–118), the French National Committee for Computerized Databases (CNIL-127020), and by EC/CCTIRS (13–118/12319). In accordance with the French regulation authorities, given the observational retrospective nature of the study, families were informed about the study by their physician including the possibility to decline participation but were not required to give written informed consent.

### 2.5. Statistical Analysis

The results are expressed as numbers and percentages for categorical variables and as median (25th quartile; 75th quartile) for continuous variables. The distribution of variables was analyzed using the chi-square test for categorical variables and the Mann–Whitney test for continuous variables.

Entry into the study cohort was defined by the date of the birth. Data were censored at the date of 5 years or last follow-up in 2014/2015 or death. Assessment of overall survival and survival without specific SCD-related complications were generated by the time to first event. Kaplan–Meier survival estimates and the 95% confidence interval (95% CI) were calculated for all SCD-related complications. Given that patients with SS or SB° tend to have a more severe clinical course than those with SC or S-beta^+^thalassemia (SB^+^), we stratified the analysis into two groups, SS/SB° (including SDPundjab) and SC/SB^+^ (including HbSE) patients, whenever appropriate. Survival curves were compared using the log-rank test. Statistical analyses were performed using STATA version 14.1 (College Station, TX 77845, USA). Overall and age-specific rates of mortality and stroke were calculated as the number of deaths/strokes divided by total person–years at risk.

## 3. Results

### 3.1. General Results

**Figure 1:**
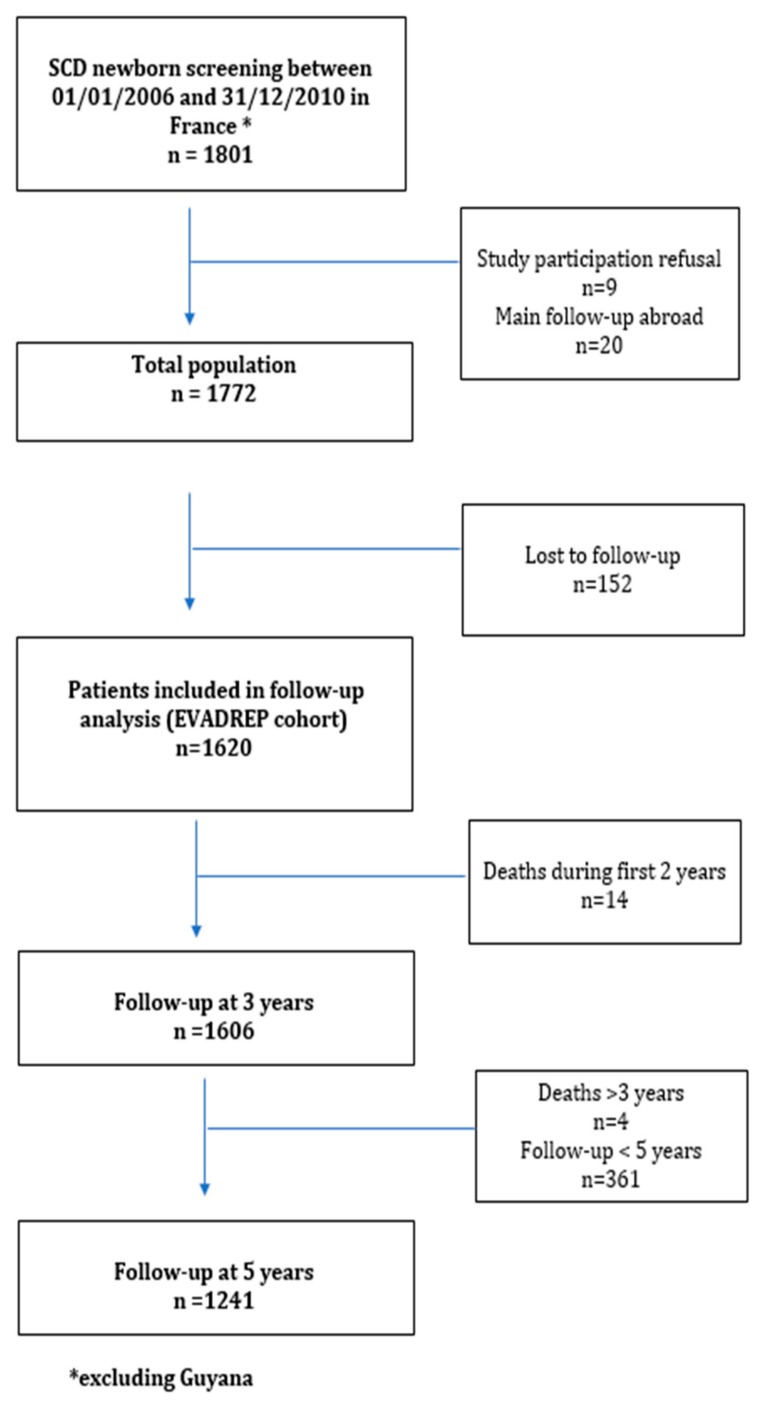
Flow-chart of the study.

Between 01/01/2006 and 31/12/2010, 1801 consecutive newborns with SCD were identified by the National NBS program (Figure 1). Nine families declined participation and 20 patients did not reside in France (their follow-up was occasionally performed in France). Out of the 1772 remaining patients, 152 (8.5%) were lost to follow-up at the time of data collection (from January 2014 to December 2015), of whom 44 were known to have returned to their country of origin. Among the 152 children lost to follow-up, 43 (28.3%) were lost to follow up following NBS and 109 (71.7%) later on. Finally, a total of 1620 patients with available follow-up data during 2014/2015 or deceased before five years were thereafter analyzed and constituted the EVADREP cohort. Subsequently, at the time of data analysis, 100% of living patients in the cohort had reached at least three years of age while 77% had reached five years.

Of note, the targeted nature of the NBS program in mainland France during our study period most likely did not leave out a significant number of SCD cases: A large retrospective study over the period ranging from 2005–2017 found 24 missed cases, representing 0.6% of the screened cohort during that same period (Pluchart and Remion, personal communication, manuscript in revision). In addition, during our study period (2006–2010), the mean national proportion of babies targeted for SCD at birth in mainland France was 30%, a rather large proportion.

A majority of children (71.8%) was diagnosed with SS or SB° and 20.3% with SC or SB^+^ disease. Main characteristics of the cohort are shown in Table 1.

Follow up was performed in 69 centers across four main French regions: Paris area, province, French West Indies (Martinique-Guadeloupe-St Martin), and Réunion-Mayotte. Over a half of patients (59.6%) were residents in the greater Paris area, while 9.4% resided in the French West Indies where a substantially greater fraction of patients had SC disease (42%).

Among the 69 centers, a majority (42; 60.8%) followed less than 20 patients included in the study: 17 (24.6%) centers followed between 20–50 patients; 10 centers (14.5%) followed more than 50 patients (between 50–100 and >100 patients in 8 and 2 centers, respectively).

### 3.2. Follow-up Results

Altogether, among the patients included in EVADREP cohort, 1540 (95.1%) had a regular follow-up while 80 (4.9%) were temporarily lost to follow-up at some point during the study period—during the first 6 months in 31(1.9%) or later during at least a 2 year period in 49 (3%). These figures did not change significantly across regions of birth, but did according to the size of the centers, the largest centers having the lowest percentage of patients lost to follow up, both following NBS or thereafter (Table 2). In addition to a hospital-based follow-up in the 69 centers, a vast majority of these children (1151, 83.6%) had a community-based follow up by the French primary care system and 1396 (91.8%) had also an identified general practitioner or pediatrician.

### 3.3. Outcomes

#### 3.3.1. Major Severe Outcomes and Prophylactic Coverage

• Survival and mortality rates and causes

**Figure 2:**
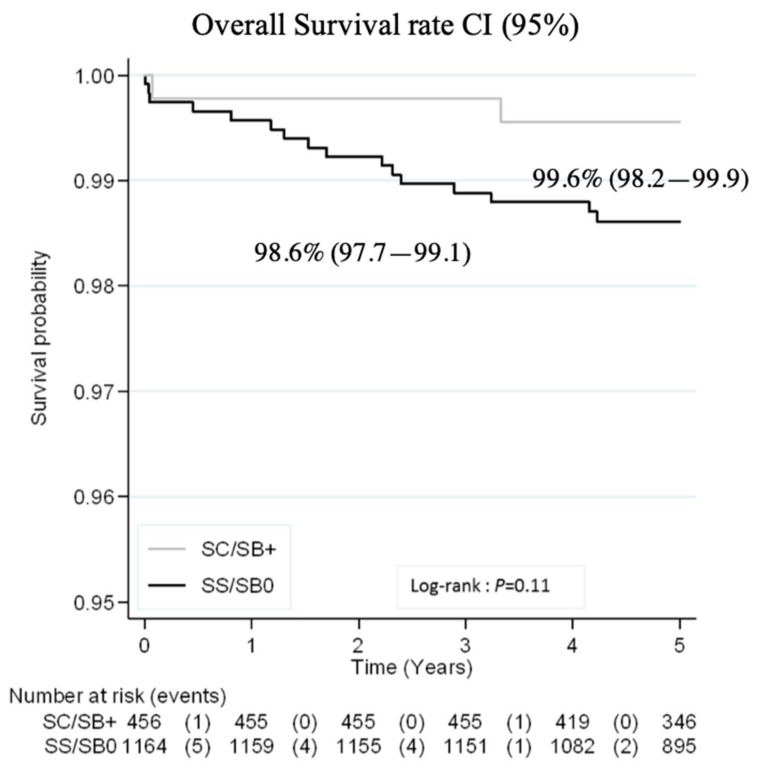
Probability of survival by five years of age.

Probability of survival for all children at five years was 98.9% (95%CI: 98.2–99.3) and was 98.6% (97.7–99.1) and 99.6% (98.2–99.9) in SS/SB° and SC/SB^+^ patients, respectively (Figure 2).

Mortality rate for all patients was 0.31/100 person–years (0.17–0.58) and 0.23/100 person–years (0.15–0.37) at two years and at five years, respectively. When analyzing by disease severity, mortality rate was 0.29/100 person–years (0.18–0.47) at 5 years in children with SS/SB° as opposed to 0.09/100 person–years (0.02–0.36) in SC/SB^+^ children.

A total of 18 patients died at a median age of 1.6 years (range: 0.01–4.2) with 10 of these deaths occurring before age 2. Among all deaths, 12 (66.7%) were SCD-related, all but one in patients with SS/SB° (Table 3). Over half of those (7/12) were from infection (four of which from pneumococcal infection). Survival at three years and probability of survival at five years excluding SCD-unrelated cause were 99.7% (95%CI: 99.3–99.9) and 99.2% (95%CI: 98.7–99.4). There was no additional death reported by the National Death Registry during the study period.

• Stroke and TCD coverage

**Figure 3:**
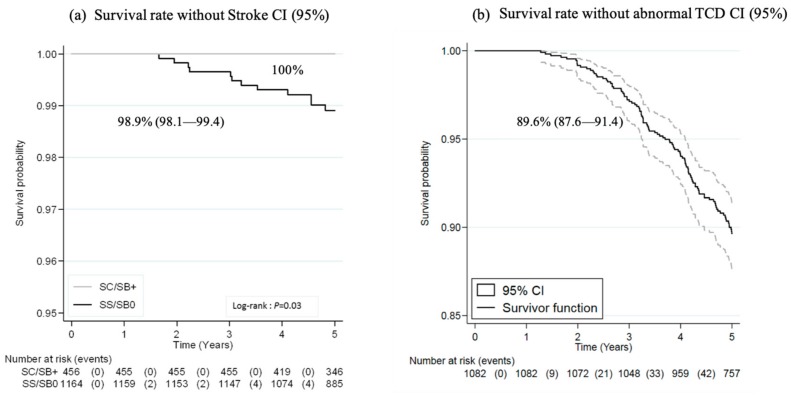
(**a**) Probability of survival without stroke; (**b**) probability of survival without abnormal transcranial Doppler (TCD) in children with SS/SB° disease.

Probability of overt stroke by five years of age was 1.1% (0.6%–1.9%) in SS/SB° patients only, as illustrated in Figure 3a and incidence rate of stroke at five years was 0.22/100 person–years (0.12–0.38). Twelve patients experienced overt stroke at a median age of 3.1 years (interquartile range (IQR), 2.2–4.3). All had an SS genotype and six had never been investigated by TCD before the occurrence of stroke. Among the remaining six patients who had undergone at least one prior TCD examination (performed at a median age of 2.1 years (1.8–2.2)), four patients had at least one normal TCD, one had a conditional TCD, and one had twice had an inconclusive TCD examination (the child was restless). Following stroke, all 12 patients were chronically transfused.

Overall, the very first TCD screening in the SS/SB° population (*n* = 1164) was performed before two and three years of age in 56% and 81% of patients, respectively. A total of 81 (7%) children had not benefitted from TCD screening at five years, with comparable numbers of unscreened children across regions. However, the proportion of unscreened children varied according to the center size with the lowest% of unscreened children in the two largest centers (5 (2.4%) versus 15 (16%) in the smallest centers) (Table 2). The median number of annual TCD in children above the age of 2 was 0.8 (0.5–1).

The overall probability of abnormal TCD (velocities ≥200 cm/s) at five years of age was 10.4% (8.6–12.4). This probability varied across regions with the highest probability in the Paris area (13.3% (10.8–16.2)) and was significantly higher in the two largest centers (20.0% (15.0–26.5)) (Table 2). The probability of abnormal TCD did not vary significantly during the study period. Probability of survival without abnormal TCD is shown in Figure 3b.

• Severe Infection and infection prophylaxis coverage

A severe infection (meningitis or septicemia) developed in 26 patients at a median age of 2.3 years (1.3–2.6), in all but two patients with SS/SB° disease. Bacteria was identified in 21 patients, with Streptococcus pneumoniae in nine patients (including one patient with SC disease). Of them, 8/9 had been fully vaccinated with PCV7 and 3/5 aged over two years with 23-valent pneumococcal polysaccharide vaccine (P23).

The outcome of severe infection was fatal in eight cases (30.8%), following pneumococcal infection in half the cases.

Overall probability of severe infection at five years was 1.6% (1.1%–2.4%) in children with SS/SB° while it was significantly lower 0.4% (0.1–1.8) in patients with SC/SB+.

• Penicillin prophylaxis and vaccination coverage

Over 99% of all SCD patients had been prescribed prophylactic penicillin, which started at a median age of 2.2 months of life (1.7–3.0). Regarding vaccination, 87% of children received at least three doses of either PCV7 or PCV13 during their first year of life. However, only 768 (47.4%) children had been fully vaccinated before age 3, i.e., had received at least four PCV and one P23. P23 was administered in 65% of patients before three years and the probability of receiving P23 was of 90% at five years. The pneumococcal immunization coverage increased according to the size of the center, ranging from 24.8% to 78.8% in centers with <10 patients to centers >100 patients (Table 2). Conversely, this coverage did not vary according to SCD genotype (data not shown).

#### 3.3.2. Other SCD-Related Events:

**Figure 4:**
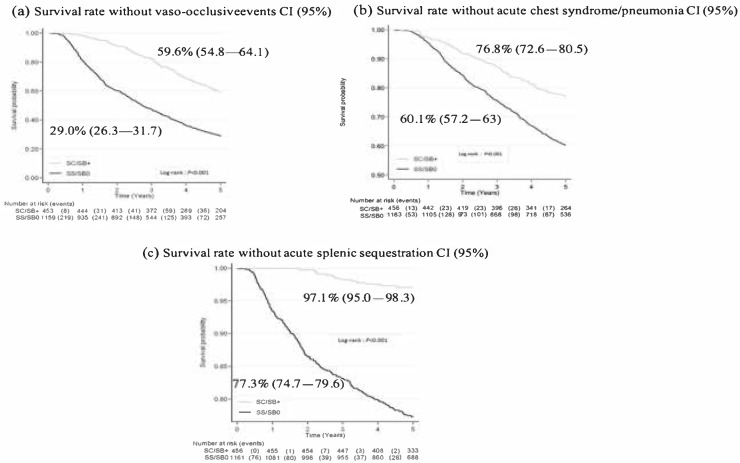
Probability of survival without (**a**) vaso-occlusive events, (**b**) acute chest syndrome/pneumonia, (**c**) acute splenic sequestration.

At three years, 42.9% of the EVADREP cohort had experienced a first VOC and the probability of survival without VOC was 37.6% (35.2–40.0) at five years. Expectedly, this probability differed significantly according to genotypes and was 29.0% (26.3–31.7) and 59.6% (54.8–64.1) in SS/SB° and SC/SB^+^, respectively, illustrated in Figure 4a.

A total of 549 children developed at least one episode of either pneumonia or ACS or both leading to a five-years probability of survival without experiencing a first episode of pneumonia/ACS of 64.8% (62.4–67.2). This probability was significantly higher for patients with SC/SB+ genotype than for those with for SS/SB° (*p* < 0.001); see also Figure 4b.

Among SS/SB° patients, 16.9% had experienced a first episode of acute splenic sequestration at three years and their probability of survival without SSA at five years was 77.3% (74.7–79.6). This complication was fatal in two cases. This probability differed significantly (97.1% (95–98.3)) in patients with SC/SB^+^ disease, as shown in Figure 4c.

When pooling all SCD-related events (death, stroke, severe infection, VOC, acute anemia, as well as abnormal TCD, pneumonia, and/or ACS and transfusion), 58.6% of children had experienced at least one event at three years. The probability of survival without SCD-related events at five years was 10.7% (8.9–12.6) for SS/SB° patients versus 46.3% (41.5–51) for SC/SB+.

At three years, 78.1% of all children had experienced a first hospitalization and the probability rose to 89.9% (88.3–91.3) by five years of age.

#### 3.3.3. Disease Modifying Therapy and Other Therapeutic Measures

• Transfusion

The probability of being transfused at least once at five years was 65.2% (62.3–68.0) in SS/SB° patients and 11.8% (9.1–15.2) in patients with SC/SB^+^ genotypes (Table 4). Splenectomy/cholecystectomy: In SS/SB° children, the probability of splenectomy was 7.0% (5.6–8.7) by five years as opposed to 0.3% (0.04–1.9) by five years in children with other genotypes (no children with SC/SB^+^ disease was splenectomized at three years). Cholecystectomy concerned only children with SS/SB° with a probability of 3.7% (2.7–5.1) by five years of age.

• Chronic transfusion:

Overall, the probability by five years of age to have benefitted from a chronic TF program was 13.2% (11.6–15) and concerned a total of 204 patients, all but one with SS/SB°. Indication for initiation of the first TF program in SCA patients was stroke in 10 cases (4.9%); acute splenic sequestration in 75 (36.8%); abnormal TCD in 76 (37.2%), and other reasons in 42 (20.6%), including vaso-occlusive events in 22 cases, additional neurovascular reasons in 14, and severe anemia in 6. At the time of data collection, the TF program was ongoing in 101 (49.5%) patients and had been halted for the remaining 103 children. Out of 60 children who were initially transfused for acute splenic sequestration and discontinued, 39 (65%) were thereafter splenectomized; among 23 children who discontinued TF initiated for abnormal TCD, 12 were thereafter switched to HU, 4 underwent HSCT, and 5 had no specified subsequent treatment. Among 15 who stopped TF initiated for ACS/VOC, 14 were switched to HU, while the last patient had no subsequent treatment. The remaining five children had miscellaneous indications for both initiating and stopping TF. The mean duration for those in whom the program was discontinued was 1.1 year (0.7–1.9). Probability of receiving a first transfusion program by five years of age was significantly increased in Paris area versus other regions and significantly increased in the largest centers (Table 2 and Table 4).

• Hydroxyurea (HU):

In children with SS/SB°, the proportion of treated patients by three years of age was 1.9% (1.3–2.9) and the probability was 13.7% (11.7–15.9) at five years. No patient with SC/SB^+^ disease was treated with HU. At five years, among the 145 children who were initiated on HU, 137 (94.5%) were still on treatment. There was no major difference in the proportion of treated children according to regions, but there was a significant difference according to the size of the centers, with an increased probability in the largest centers (Table 2 and Table 4).

• Hematopoietic stem cell transplantation (HSCT):

Only nine patients with SS/SB° underwent HSCT before the age of five, at a median age of 4.1 (4.0–4.4). Indication for HSCT was alloimmunization in one case, VOC despite HU in one case, acute splenic sequestrations in one case, and cerebral vasculopathy in six cases (abnormal TCD in five patients).

## 4. Discussion

This study allows for the first time, to the best of our knowledge, a “real world” analysis on a national scale of both the early burden and mortality of sickle cell disease in a high-income country where up-to-date standard of care is easily available. Outcomes regarding 1620 children born between 2006 and 2010 and evaluated at five years show a very low SCD-related mortality and demonstrates that frequency of serious SCD-related complications such as severe infection and stroke have drastically declined. In contrast, the overall burden of the disease remained very high with a sustained elevated proportion of abnormal TCD results, VOC, ASS, ACS, transfusion requirement, hospital admissions, and chronic transfusion programs, contrasting sharply with a very low fraction of children benefitting from disease modifying or curative therapy, namely hydroxyurea and HSCT. Overall, 8.5% of the children identified following NBS were lost to follow-up at the time of data collection, with a fraction of them known as not residing in France. In this study, overall mortality rate for all patients was 0.23/100 person–years during the first five years of life and overall probability of survival was of 98.9%. Although the rate of death between 1–4 years of age remained higher than in the French general population for this age-group during the same period (0.19 versus 0.03/100), [12] these results align with a recent UK national study [8] that showed a reduced mortality in children under five (death rate of 0.17/100 person–years of follow-up for all sickle cell disorders and 0.26 per 100 person–years in homozygous children) or older reports from pediatric cohorts in similar high-income settings (Dallas cohort, 1983–2007, 0.52/100 patient–years [6], New York, 2000–2008, 0.38/100 person–years in the first two years of life) [13], East London (1982–2005, no deaths at five years) [5] or regional French cohorts (0.25 and 0.32/100 patient–years, respectively in North East Paris area and Créteil) [4,7]. Arguably, our study differs by focusing on the first five years of life only, but in SCD these are the most vulnerable years, along with young adulthood when a second peak of mortality occurs after transitioning [14]. Residual risks of stroke by five years of age was low (1.1%) in the range found in children benefitting from early TCD screening (0.7%–1.9%) [4]. Likewise, probability of severe infection (meningitis or septicemia) was low (1.6%), despite a known persistent increased risk of invasive pneumococcal infection with a high mortality rate in SCA patients [15,16]. This low rate probably reflects the effectiveness of pneumococcal vaccination and penicillin prophylaxis, as reported consistently in other settings [17]. Indeed, vaccination coverage regarding conjugated vaccine was 87% during first year of life, although this percentage fell to 47.4% by age three regarding full pneumococcal vaccination including P23 at two years, mainly because P23 was generally administered later i.e., during the fourth or fifth year of life. In comparison, vaccination coverage, which included both PCV and P23, was 64.3% at five years in a state study in children benefitting from Medicaid in the US [18]. Almost all infants in the EVADREP cohort benefited from early prophylactic penicillin initiated within three months of NBS, a figure comparable to a nation-wide report in England, [8] although there was no further information on compliance issues in either studies. Regarding TCD screening, a vast majority of children at risk (80.5%) were investigated with TCD before three years of age and the probability rose to 93% by age 5, contrasting sharply with cross sectional reports of TCD annual screening rates ranging from 22% to 44% in the US [19].

In addition to the known beneficial effect of current management guidelines, these results also reflect a real-life efficient access to medical care for patients in the French healthcare system, including “vulnerable” populations like patients with SCD who are, for the majority, children of recently immigrated families with significant socio-economic difficulties [7]. In France, access to care for children under six is highly facilitated through a network of primary medical care where free follow-up and immunization is offered. Additional specific prophylactic and therapeutic measures for SCD children are free of charge once the diagnosis is confirmed, regardless of age or the region of follow-up. Centers are organized in a national network where patients may easily access emergency departments and may be addressed to tertiary hospitals for expertise if necessary. In these conditions that actually allow for the practical application of guidelines, there was indeed an overall very good implementation of recommendations.

Despite these favorable conditions, it is noteworthy to mention that 6 of the 12 strokes occurred in children who had not been screened. Likewise, severe infections (including pneumococcal) still accounted for the main cause of death. Although severe pneumococcal infections most often occurred in patients fully vaccinated with PVC as previously reported, [17] further improvement can be achieved by increasing P23 vaccination coverage. One striking and recurrent result was the somewhat counter intuitive finding of decreased TCD and vaccination coverage in smaller centers, along with higher rates of patients lost to follow-up. It could indeed be hypothesized that with fewer patients, the coverage would be better. In fact, it is probable that not only do larger centers have specialized staff to ensure standard of care surveillance, but also dedicated time to track down non-attenders and reschedule missed appointments. Smaller centers, conversely, deal with pediatric patients with much more variable medical conditions, are probably less focused on specific needs, and are not staff-equipped to follow-up on compliance or educational issues. In addition, TCD screening requires specialized trained physicians and is generally performed in larger centers at farther distance from the patient’s residence, a factor that was shown to negatively influence TCD utilization rates [20].

This study showed that 8.4% of children screened at birth were lost to follow-up in their first five years of life at the time of data collection. While there is no possibility of speculating on the outcomes, because this a nation-wide study, it is probable that none of these children died nor had a severe event such as stroke or severe infection in France. While such events may still have occurred abroad, it is probable that mortality and severe morbidity rates were not significantly biased by this missing data. Although the rate of patients lost to follow-up is similar on a national scale to results from regional or single center European cohorts, which range from 6%–11% [4,7], the figure remains significant and calls for further improvement in order to decrease the number of non-attenders.

Results on less severe outcomes such as abnormal TCD, VOC, ASS, ACS, and hospitalization rates showed a very high burden of the disease with no improvement in their frequency when compared to cohorts in the previous decade [5]. The overall probability of abnormal TCD at five years was 10.4%, comparable to 7% in the East London cohort but lower than in regional French cohorts from tertiary centers (around 20%), [4,5] a difference that may reflect the “large center effect” with presumably a higher proportion of severe patients referred for tertiary expertise and/or monitored more intensively. Nearly 2/3 of children had experienced a VOC by five years and the same proportion (65.2%) was transfused at least once before age 5. The probability of an SCD-related event and the rate of first hospitalization were very high (79.3% and 89.9%, respectively, at five years). In sharp contrast, disease-modifying or curative therapies were only given to a small fraction of patients: HSCT was performed in only nine children while hydroxyurea therapy was prescribed in only 10%. More classical therapy like chronic transfusion programs was, on the other hand, widely used since 13% of the cohort was treated at least temporarily with a chronic transfusion program before five years. Arguably, this cohort was born in 2006–2010, i.e., before the publication of HU trials in infants [21] and the extension of indication as in the US to all children above nine months regardless of clinical severity [22] or as an alternative to transfusion in selected children with abnormal TCD [4,23]. In addition, HU was and still is licensed in France for children over two years with severe vaso-occlusive symptoms. It is likely that in the upcoming years, the proportion of young children treated with HU (or additional drugs) will increase. Likewise, the very small number of children who benefitted from HSCT during the study period probably reflects the relative reluctance of SCD physicians in addressing very young patients to transplant if they have not experienced severe complications, particularly without prior HU treatment. Recent studies have shown significant improvement with time of long-term results following matched-sibling-donor stem cell transplantation offering 97.8% event-free survival at five years post-transplant among 190 patients transplanted after year 2000 [24]. In addition, outcomes are improved when HSCT is performed at a younger age [25]. This should further encourage HSCT in SCD, given the high burden of the disease.

## 5. Conclusions

In conclusion, this study shows, on a national scale, the benefits of NBS and preventive measures, confirms the good implementation of these measures, and does not reveal major disparity across regions or centers, although the proportion of severe patients is highest in larger centers, most often located in the Paris area. Notwithstanding, this study underscores the importance of referral of patients to tertiary centers where systematic annual specialized work-up may be more readily performed and general educational or immunization issues double-checked by dedicated healthcare providers in order to further improve vaccination and TCD coverage, notably. In addition, this study reveals a very low proportion of children treated with HU or HSCT despite a sustained burden of the disease. Predictably, future studies will demonstrate an increase in disease modifying therapy use. In addition, given the ongoing progress in HSCT outcomes, indications will broaden so that altogether curative treatments (potentially including gene therapy [26]) will hopefully benefit more children.

## Figures and Tables

**Table 1:** General characteristics of the population.

	Total	Paris Area	Province	French West Antilles	Mayotte-Réunion
*n* (%)	1620 (100)	967 (59.6)	443 (27.3)	152 (9.4)	58 (4.4)
SS/SB°/SDPunjab	1164 (71.8)	703 (72.7)	336 (75.8)	74 (48.7)	51 (87.9)
SC/SB^+^/Other *	456 (28.2)	264 (27.3)	107 (24.2)	78 (51.3)	7 (12.1)
Sex ratio (M/F)	824 (50.9)	-	-	-	-

* SO-Arab/SE/S-Lepore.

**Table 2:** Outcomes and coverage rates by five years of age according to center size.

Size of Center †	Total	<10	(10–20)	(20–50)	(50–100)	≥100	*p* Value *
Nb. of centers	69	24	18	17	8	2	-
Nb. of patients	1620	125	258	452	516	269	-
Nb of patients with continued follow-up, *n* (%)	1540 (95.1)	116 (92.8)	247 (95.7)	421 (93.2)	490 (94.9)	266 (98.9)	0.009
No TCD screening *N*, (%) **	81 (7.0)	15 (16.0)	18 (9.4)	25 (7.1)	18 (5.6)	5 (2.4)	<0.001
Complete pneumococcal coverage ^#^ *N*, (%)	768 (47.4)	31 (24.8)	89 (34.5)	206 (45.6)	230 (44.6)	212 (78.8)	<0.001
Probability of HU treatment ** *n*, %	145 13.7% (11.7–15.9)	14 16.5% (10.1–26.4)	28 16.5% (11.7–23.1)	27 8.4% (5.8–12)	35 12.1% (8.8–16.4)	41 21.2% (16–27.7)	<0.001
Probability of TF program ** n, %	204 18.4% (7.2–20.8)	10 11.2% (6.2–19.8)	27 15.0% (10.5–21.2)	39 11.6% (8.6–15.6)	56 18.2% (14.3–23)	72 36.3% (30–43.5)	<0.001
Probability of abnormal TCD ** n, %	105 10.4% (8.6–12.4)	8 10.5% (5.4–20.0)	19 11.5% (7.5– 17.4)	21 6.8% (4.5-10.3)	19 7.0% (4.5–10.8)	38 20.0% (15–26.5)	<0.001

† Size of center according to the number of patients enrolled in the study * Log-rank test or chi-squared test; ** only for SS/SB° patients ^#^ defined by ≥4 doses of pneumococcal conjugated vaccine (either PCV7 or PCV13) and 1 polysaccharidic dose at 3 years; TCD: Transcranial Doppler; TF: Transfusion.

**Table 3:** Overall causes of death.

Causes of death (*n* = 18)
Unrelated causes: *n* = 6
Pulmonary dysplasia
Spinal muscular atrophy
Neonatal herpes
Premature birth
Mitochondriopathy
Neonatal Streptococcus B meningitis
SCD-related infectious causes: *n* = 7
Pneumococcal septicemia (*n* = 3)
Pneumococcal meningitis
Undocumented sepsis (*n* = 3)
Miscellaneous SCD-related causes: *n* = 5
Dehydration
Acute pancreatitis
Acute cardiorespiratory failure
Acute splenic sequestration *n* = 2

**Table 4:** Proportion of therapeutic intervention or disease modifying therapy initiation by three years of age and probability by five years of age.

	All	SS/SB°	SC/SB^+^	*P* Value †
**First transfusion** by 3 years by 5 years	32.8% (30.6–35.2) 50.1% (47.6–52.6)	43.8% (41.0–46.2) 65.2% (62.3–68.0)	4.8% (3.2–7.3) 1.8% (9.1–15.2)	<0.001
**Splenectomy** by 3 years by 5 years	1.9% (1.4–2.7) 5.1% (4.1–6.3)	2.7% (1.9–3.8) 7.0% (5.6–8.7)	0 0.3% (0.04–1.9)	<0.001
**Cholecystectomy** by 3 years by 5 years	0.1% (0.03–0.5) 2.7% (2.0–3.7)	0.2% (0.04–0.7) 3.7% (2.7–5.1)	0 0	<0.001
Chronic Transfusion program * by 3 years by 5 years	7.0% (5.9–8.4) 13.2% (11.6–15.0)	9.7% (8.1–11.6) 18.4% (16.2–20.8)	0.2% (0.03–1.6) 0.2% (0.03–1.6)	<0.001
**Hydroxyurea** by 3 years by 5 years	1.4% (0.9–2.1) 9.8% (8.4–11.5)	1.9% (1.3–2.9) 13.7% (11.7–15.9)	0 0	<0.001

* A chronic transfusion program was defined by a duration > 3 months, † log-rank test for comparison of SS/SB° versus SC/SB+ patients at 5 years.
